# Nerve Stimulator versus Ultrasound-Guided Femoral Nerve Block; a Randomized Clinical Trial

**Published:** 2017-01-18

**Authors:** Arash Forouzan, Kambiz Masoumi, Hasan Motamed, Mohammad Reza Gousheh, Akram Rohani

**Affiliations:** 1 Department of Emergency Medicine, Ahvaz Jundishapur University of Medical Sciences, Ahvaz, Iran.; 2 Department of Anesthesiology, Ahvaz Jundishapur University of Medical Sciences, Ahvaz, Iran.

**Keywords:** Femoral fractures, ultrasonography, interventional, transcutaneous electric nerve stimulation, pain management, emergency service, hospital

## Abstract

**Introduction:**

Pain control is the most important issue in emergency department management of patients with femoral bone fractures. The present study aimed to compare the procedural features of ultrasonography and nerve stimulator guided femoral nerve block in this regard.

**Method:**

In this randomized clinical trial, patients with proximal femoral fractures presenting to emergency department were randomly divided into two groups of ultrasonography or nerve stimulator guided femoral block and compared regarding success rate, procedural time, block time, and need for rescue doses of morphine sulfate, using SPSS 20.

**Results:**

50 patients were randomly divided into two groups of 25 (60% male). The mean age of studied patients was 35.14 ± 12.95 years (19 – 69). The two groups were similar regarding age (p= 0.788), sex (p = 0.564), and initial pain severity (p = 0.513). In 2 cases of nerve stimulator guided block, loss of pinprick sensation did not happen within 30 minutes of injection (success rate: 92%; p = 0.490). Ultrasonography guided nerve block cases had significantly lower procedural time (8.06 ± 1.92 vs 13.60 ± 4.56 minutes; p < 0.001) and lower need for rescue doses of opioid (2.68 ± 0.74 vs 5.28 ± 1.88 minutes; p < 0.001).

**Conclusion:**

Ultrasonography and nerve stimulator guided femoral block had the same success rate and block duration. However, the ultrasonography guided group had lower procedure time and lower need for rescue doses of morphine sulfate. Therefore, ultrasonography guided femoral block could be considered as an available, safe, rapid, and efficient method for pain management of femoral fracture in emergency department.

## Introduction

Femoral bone fractures are not infrequent and are usually associated with severe pain (1, 2). Pain control is the most important issue in emergency department management of these patients. Different methods of pain management such as administration of intravenous opioids, tranquilizers and muscle relaxants, and even inhaled drugs are introduced for this propose (3, 4). However, allergic reactions, airway compromise, respiratory depression, and hypotension are among the most important complications of the mentioned methods. 

Regional nerve blocks have shown benefits over the procedural sedation and analgesia (PSA) for pain management in emergency settings (5-8). Regional anesthesia without any important adverse effects on central nervous system or systemic circulation, could be helpful in managing pain and decreasing the volume of narcotic and opioid usage (1, 9). 

Anatomic landmarks guided femoral nerve block is an effective method for reducing pain in adults and children referring to emergency departments with femoral fractures (6). However, injection of drug in the femoral artery is the most undesirable complication of this method. 

Nowadays, nerve stimulator and ultrasonography guided methods of block have increased the safety level of the procedure (5, 10). Ultrasonography allows physicians to observe the nerves directly so that the needle can be kept away from sensitive organs and distribution of regional anesthetic can be monitored. Also, using transcutaneous electric nerve stimulation could be helpful in localizing the nerve and increasing the effectiveness of block (11, 12). 

However, emergency physicians are more familiar with ultrasonography than nerve stimulator, and ultrasonography as a noninvasive tool is more available in emergency departments. Based on the above mentioned points, the present study aimed to compare the characteristics of ultrasonography and nerve stimulator guided femoral nerve block in pain management of femoral fractures in emergency department.

## Methods

***Study design and setting***

This randomized clinical trial was conducted on patients with proximal femoral fractures (including neck, inter-trochanteric, and proximal shaft fractures), admitted to emergency departments of Imam Khomeini Hospital, Ahvaz, Iran, from January to December 2015, aiming to compare the procedure features of nerve stimulator and ultrasonography guided femoral nerve block techniques. The protocol of the study was approved by ethical committee of Ahvaz Jundishapur University of Medical Sciences and registered in Iranian registry of clinical trials under number IRCT2015030221289N1. Researchers adhered to all principles of Helsinki declaration and confidentiality of patients’ information during the study period. Informed consent was obtained from the study subjects before enrollment. 

***Participants***

Patients with proximal femoral fractures, aged 18-80 years, referred to emergency department were included. The exclusion criteria were hemodynamic instability, loss of consciousness, contraindications of receiving regional anesthesia or opioid administration (hypersensitivity to any variety of regional anesthetics such as amide and other compounds, systemic or local infections, abnormal neurological examination, and risk of compartment syndrome), opioid addiction, severe pulmonary or heart disease, diabetes, and coagulopathy.

***Intervention***

Eligible patients were randomly divided into two groups of nerve stimulator or ultrasonography guided femoral nerve block, using simple random sampling technique. All patients received 0.1 mg per kg intravenous morphine sulfate before initiation of procedure. A single dose of 10 mL of lidocaine1% was used for regional anesthesia. 

Nerve stimulation was done by a 50 gauge needle (with 20 degrees tip angle) using a Pajunk multistim sensor device. The needle was inserted with a 45 degrees angle just inferior and lateral to where the femoral artery crosses the inguinal ligament. At first, the flow rate of device was set at 2.5 mV, and then after an appropriate response from the muscle (quadriceps muscle contraction), the flow rate was reduced to 0.4 mV so that the muscle response could still be visible. 

**Figure 1:**
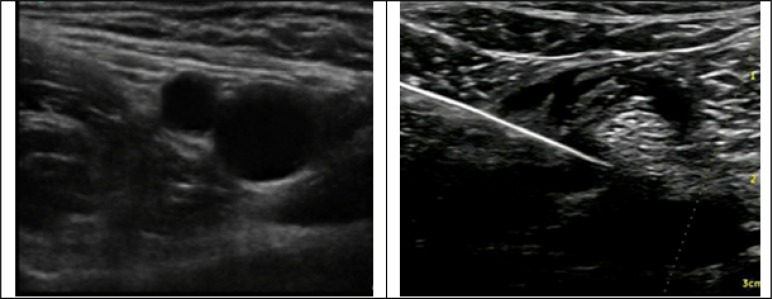
Ultrasonography view of right inguinal structures

**Figure 2:**
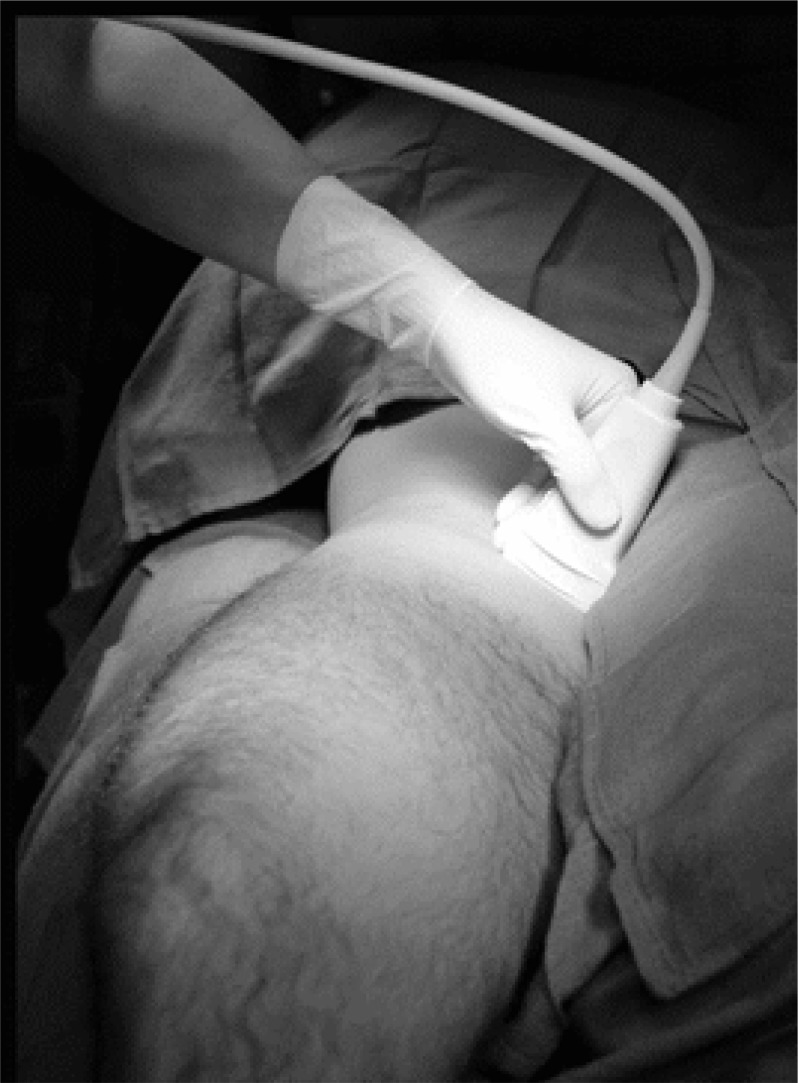
Position of ultrasonography probe in inguinal area

Ultrasonography guided nerve blocks were done using a high frequency (7 – 12 MHz) linear array probe (The SonoAce-X8 Ultrasound system -Samsung Medison Co., Ltd., South Korea) in a supine position with totally abducted legs (figure 1 and 2). 

All patients were under continuous cardiac, pulse rate, respiratory rate, blood pressure, and O_2_ saturation monitoring during the procedure. Pain severity was measured using visual analogue scale (VAS). Considering 30 minutes duration for loss of pinprick sensation (13), in cases with ≥ 3 pain score, 30 minutes after block, additional rescue doses of morphine sulfate (0.1 mg/kg) were administered.

The sensory (pinprick sensation) and motor response were measured every 5 minutes during 30 minutes after the injection of lidocaine. Leg extension against gravity and passive hip flexion in 45° were measured for motor nerve block examination. 

Procedures were done by a trained senior emergency medicine resident under supervision of an emergency medicine specialist. Operators were trained regarding ultrasonography and nerve stimulator guided nerve block during an 8 hour educational course and by doing the procedure under supervision of an expert radiologist.

***Data gathering***

A predesigned checklist consisting of demographic data (age, sex), initial pain severity, procedure time, block duration, success rate, and need for rescue doses of morphine sulfate was used for data gathering. Procedure time was defined as interval between lidocaine injection and loss of pinprick sensation. Also, interval between loss and recovery of pinprick sensation was considered as block duration. A successful block was defined as complete sensory loss in the femoral nerve distribution by 30 minutes. Data gathering was done by a blinded observer.

***Statistical analysis***

Considering 1.2 and 0.4 mg rescue doses of morphine sulfate in the two groups (14), 95% confidence interval, and the power of 80%, the number of samples per arm was estimated to be 25 cases. Analysis was done using SPSS 20. Data were reported as mean and standard deviation or frequency and percentage. T test was used to compare means and chi square or Fisher’s exact test for comparing the categorical variables. P-value <0.05 was considered as significant. 

## Results

50 patients with proximal femur fracture were randomly divided into two groups of 25 (60% male). The mean age of studied patients was 35.14 ± 12.95 years (19 – 69). Table 1 compares the baseline characteristics of studied patients. Two groups had the same condition regarding age (p= 0.788), sex (p = 0.564), and initial pain severity (p = 0.513).

Loss of pinprick sensation within 30 minutes of injection did not happen in 2 cases of nerve stimulator guided block (success rate: 92%). The success rate, mean procedure time, block time, and amount of rescue doses of morphine sulfate, which were used, were compared between two groups in table 2. Ultrasonography guided nerve block cases had significantly lower procedural time (p < 0.001) and lower need for rescue doses of opioid (p < 0.001). 

## Discussion

Based on the main findings of the present trial, ultrasonography and nerve stimulator guided femoral block had the same success rate and block duration. However, the ultrasonography guided group had lower procedure time and lower need for rescue doses of morphine sulfate. 

Kumar et al., comparing these two techniques for axillary brachial plexus block, also showed the similar success rate (95% versus 93.2; p = 0.35) of both groups (15). In another study by Cataldo et al., the failure rates after 30 minutes in both groups were not significant (16). 

In consistency with our findings, Tran et al. (17), demonstrated that the procedure time of superficial cervical plexus block was lower in the ultrasonography guided nerve block group (119 vs. 61 seconds, P<0.001). Duration of procedure did not show any difference between the two methods. 

Kumar et al., (15) showed that the duration of sensory axillary nerve blocks in the ultrasonography guided group was 6.33 minutes versus 6.17 minutes in the nerve stimulation group. Durations of motor block in the ultrasound-guided group and the nerve stimulation group were 23.33 and 23.17 minutes, respectively. Unlike our study, these differences were not statistically significant.

**Table 1:** Comparison of baseline characteristics between studied groups

**Variable**	**Ultrasonography**	**Nerve stimulator**	**P value**
**Age (year)**	35.64 ± 13.29	34.64 ± 12.86	0.788
**Sex**			
Male	16 (64)	14 (56)	0.564
Female	9 (36)	11 (44)	
**Pain severity (VAS)** *	8.88 ± 0.72	8.52 ± 2.63	0.513

* VAS: visual analogue scale at the initiation of procedure. Data were presented as mean ± standard deviation or frequency and percentage.

**Table 2:** Comparison of success rate, procedure time, block duration, and amount of morphine sulfate rescue doses between two groups

**Variable**	**Ultrasonography**	**Nerve stimulator **	**P value**
**Success rate (30 minute)**	25 (100)	23 (92)	0.490
**Procedure time (minute)**	8.06 ± 1.92	13.60 ± 4.56	< 0.001
**Block duration (minute)**	61.56 ± 16.50	57.64 ± 23.85	0.502
**Rescue dose (mg)**	2.68 ± 0.74	5.28 ± 1.88	< 0.001

Data were presented as mean ± standard deviation or frequency and percentage.

A randomized clinical trial performed by Perlas et al., comparing the success rate of the sciatic nerve block with ultrasonography and nerve stimulator techniques, showed that the duration of the procedure was similar in both groups (18). Rubin et al., showed that duration of the procedure and the time of onset for nerve block in the ultrasound-guided group were significantly lower than the nerve stimulation group (19). 

In a meta-analysis by Choi et al., reporting seven studies in which opioid consumption was reported, the reduction was mentioned in the ultrasound-guided method in three studies (20). In the three studies that evaluated the time of onset of analgesia, the ultrasonography guided approach was preferred. Oberndorfe et al. (21) showed that the amount of drug administration for regional anesthesia in the ultrasonography guided group was less than the nerve stimulation group (P <0.001).

In contrast, Maalouf et al. showed that the mean amount of oral morphine equivalents used in ultrasonography and nerve stimulator guided groups were similar (22). 

It seems that using ultrasonography guided femoral nerve block could be considered as an available, safe, rapid, and efficient method for pain management of patients presenting to emergency department following femoral fracture. 

***Limitation***

Low sample size and not performing the study in a double blind manner are among the most important limitations of this study. However, data gathering by a blinded observer can decrease the bias. 

## Conclusion:

Based on the main findings of the present trial, ultrasonography and nerve stimulator guided femoral block had the same success rate and block duration. However, the ultrasonography guided group had lower procedure time and lower need for rescue doses of morphine sulfate. 
